# Supplementary material for: The Extract of Scutellaria baicalensis Attenuates the Pattern Recognition Receptor Pathway Activation Induced by Influenza A Virus in Macrophages
Source: Viruses. 2023 Jul 8;15(7):1524. doi: 10.3390/v15071524 (PMC10384909; doi:10.3390/v15071524)
Supplement: Supplementary file 1 [file viruses-15-01524-s001.zip › viruses-2449315-supplementary.pdf]

Supplementary Table S1. Ultra-high-pressure liquid chromatography time-of-flight mass spectrometer (UPLC/Q-TOF MS) analysis of SBE.

No.	Component	Formula	RT(min)	Ion	Theoretical(m/z)	Measured(m/z)	Error(ppm)	MS^2(m/z)
1	Scutellarin	$C_{21}H_{18}O_{12}$	3.23	[M-H]$^-$	461.0720	461.0714	-1.30	461.0714[M-H]- ,923.151[2M-H]$^-$ 285.0406[M-H-C6H8O6]$^-$ 463.0880 [M+H]$^+$,
			3.37	[M+H]$^+$	463.0877	463.0880	0.65	287.0557 [M+H-C6H8O6]$^+$, 169.0182[M+H-C6H10O5-C8H5O]$^+$ 755.2402 [M-H]$^-$,
2	Baicalin	$C_{21}H_{18}O_{11}$	4.63	[M-H]$^-$	445.0771	445.0771	0.00	445.0771[M-H]$^-$,891.1635 [2M-H]$^-$, 269.0449 [M-H-C6H8O6]$^-$ 447.0927[M+H]$^+$,
			4.89	[M+H]$^+$	447.0927	447.0927	0.00	271.0606[M+H-C6H8O6]$^+$ 169.0145[M+H-C6H8O6-C8H6]$^+$ 103.0554[M+H-C7H4O5]$^+$
3	Chrysin-7-O-glucuronide	$C_{21}H_{18}O_{10}$	5.30	[M-H]$^-$	429.0822	429.0816	-1.40	253.0499 [M-H-C6H8O6]$^-$, 151.0242[M-H-C6H8O6-C8H6]$^-$ 431.0981[M+H]$^+$,
			5.57	[M+H]$^+$	431.0978	431.0981	0.70	255.0665[M+H-C6H8O6]$^+$, 153.0246[M+H-C6H8O6-C8H6]$^+$ 135.0240[M+H-C6H8O6-C8H6-H2O]$^+$
4	Oroxylin A-7-O-glucuronide	$C_{22}H_{20}O_{11}$	5.34	[M-H]$^-$	459.0927	459.0923	-0.87	919.1946[2 M-H]- ,459.0923[M-H]$^-$, 283.0605[M-H-C6H8O6]$^-$ 461.1086[M+H]$^+$,
			5.59	[M+H]$^+$	461.1084	461.1086	0.43	285.0768 [M+H-C6H8O6]$^+$, 255.0663 [M+2H-C6H8O6-OCH3]$^+$, 183.0237[M+H-C6H8O6-C8H6]$^+$
5	Wogonoside	$C_{22}H_{20}O_{11}$	5.63	[M-H]$^-$	459.0927	459.0927	0.00	919.1943[2M-H]- ,459.0927[M-H]$^-$, 283.0601[M-H-C6H8O6]$^-$
			5.60	[M+H]$^+$	461.1084	461.1089	1.08	461.1089[M+H]$^+$,483.0907 [M+Na]$^+$, 285.0766 [M+H-C6H8O6]$^+$, 183.1506[M+H-C6H8O6-C8H6]$^+$
6	Salvigenin	$C_{18}H_{16}O_6$	7.09	[M-H]$^-$	327.0869	327.0872	0.92	327.0872[M-H]$^-$,195.0221[M-H-C9H8O]$^-$ 329.1027 [M+H]$^+$,351.0841[M+Na]$^+$,
			7.45	[M+H]$^+$	329.1025	329.1027	0.61	197.0243[M+H-C9H8O]$^+$ 498.2899[M-H]$^-$,434.0767[M-H-SO2]$^-$,
7	Wogonin	$C_{16}H_{12}O_5$	7.72	[M-H]$^-$	283.0606	283.0606	0.00	181.0219[M-H-C6H8O6-C8H6]$^-$ 285.0760[M+H]+ ,307.0576[M+Na]$^+$,
			8.10	[M+H]$^+$	285.0763	285.0760	-1.05	255.0665[M+2H-OCH3]$^+$, 183.0246[M+H-C6H8O6-C8H6]$^+$

Supplementary Table S2. Primers for RT-qPCR used in this study.

Genes	Sequence (5'- 3')	Orientation
NP	CCAGCAAAAGCAGGGTAGATAA	Forward
NP	CCCAGTAGAAACAAGGGTATTTT	Reverse
IL-1β	TTCAGGCAGGCAGTATCACTCATTG	Forward
IL-1β	TGTCGTTGCTTGGTTCTCCTTGTAC	Reverse
IL-6	CTTCTTGGGACTGATGCTGGTGAC	Forward
IL-6	CTCTCTGAAGGACTCTGGCTTTGTC	Reverse
TLR3	CCTTGCGTTGCGAAGTGAAGAAC	Forward
TLR3	GAGAAGGAACCGTTGCCGACATC	Reverse
TLR7	AGATGTCCTTGGCTCCCTTCTCAG	Forward
TLR7	GTGGCGGTCAGAGGATAACTTGTG	Reverse
TLR8	GTGGAAATCGTCTTGACCGTTTGTG	Forward
TLR8	GGAAGCCAGAGGGTAGGTGAGAG	Reverse
RIG-I	ACGATGTCCGAGAAGCAGCATTC	Forward
RIG-I	CATCAGCGACCGAGGTAGCAATC	Reverse
MDA5	AAGAAGAAGCAGGCATCTGAATCCG	Forward
MDA5	ACACCGTCATCGTCTCCACTCTC	Reverse
NLRP3	CTCTGTTCACTGGCTGCGGATG	Forward
NLRP3	TGGTCCTTTCCTCACGGTCTCC	Reverse
NLRC4	GCGAGTCTGGCAAAGGGAAGTC	Forward
NLRC4	CCGTGGTGGTGGTGACAATGAC	Reverse
NLRC5	TGGAGATGAGGTGGCTGCTGAG	Forward
NLRC5	GTCTTGAGAAGTCCGTTGGGTTCC	Reverse
cGAS	CTTCCCAGCCTGACATTGCCTTC	Forward
cGAS	GCCACGCTTCCTGCTATGATGAC	Reverse
IFI202	CCTCAAGCCTCTCCTGGACCTAAC	Forward
IFI202	CTCTAGGATGCCACTGCTGTTGAAG	Reverse
IFI204	TGTGTTAGAGGCTGCTCCTGACC	Forward
IFI204	CCACCACTTCTATGCTTCCTGAACC	Reverse
MyD88	GATTCTCTGATGCCGTCCTGTCTAC	Forward
MyD88	AACTCCTGATTCTCCTGCCTCTACC	Reverse

MAVS	CCTCCTCGCTTCTCCACTCCTG	Forward
MAVS	TCTGCCTCTCACACTCTCACCTTAG	Reverse
ASC	GCAACTGCGAGAAGGCTATGGG	Forward
ASC	CTCATCTTGTCTTGGCTGGTGGTC	Reverse
IRF7	GGCTCGGACGCTGGATTAACAC	Forward
IRF7	CACAGGCAGTCTGGGAGAATCAAAG	Reverse
IL-10	GGACAACATACTGCTAACCGACTCC	Forward
IL-10	CTTCACCTGCTCCACTGCCTTG	Reverse
IL-15	TCAGCAGATAACCAGCCTACAGGAG	Forward
IL-15	ATGAAGACATGAATGCCAGCCTCAG	Reverse
IL-18	AGCCGCCTCAAACCTTCCAAATC	Forward
IL-18	GTCACAGCCAGTCCTCTTACTTCAC	Reverse
IFI35	CTTCACTGCCAACCCTGCTCTG	Forward
IFI35	ACTTGCCTGCCCTGCTTAGTTTG	Reverse
NMI	CACAGGCGTCAGATTCCAGGTTC	Forward
NMI	GTAAGTCCTCCCCAGTCTCCTCATC	Reverse
GAPDH	TCACCATCTTCCAGGAGCGAGAC	Forward
GAPDH	TGAGCCCTTCCACAATGCCAAAG	Reverse

Supplementary Table S3. Summary of RNA-seq data analysis.

Sample Name	Raw Reads	Raw Base	Clean Reads	Clean Base	Q20	Q30	GC content
Mock-1	57.88M	8.68G	55.39M	8.31G	97.48%	92.42%	50.45%
Mock-2	63.92M	9.59G	61.41M	9.21G	97.41%	92.24%	50.61%
Mock-3	56.26M	8.44G	53.93M	8.09G	97.41%	92.20%	50.34%
PR8-1	61.06M	9.16G	58.12M	8.72G	97.68%	92.98%	50.21%
PR8-2	43.36M	6.50G	41.42M	6.21G	96.88%	90.46%	49.86%
PR8-3	26.07M	3.91G	24.55M	3.68G	98.35%	94.68%	49.58%
SBE-1	75.28M	11.29G	71.93M	10.79G	97.69%	93.02%	50.55%
SBE-2	46.32M	6.95G	43.86M	6.58G	98.16%	94.37%	50.47%
SBE-3	61.76M	9.26G	58.83M	8.82G	97.67%	92.75%	50.16%

Mock-1, Mock-2, and Mock-3: three biological replicates of the normal cell samples; PR8-1, PR8-2 and PR8-3: three biological replicates of the PR8-infected samples; SBE-1, SBE-2, and SBE-3: three biological replicates of SBE treatment with PR8-infected samples.

Supplementary Table S4. Reference genome alignment statistics.

Sample Name	Total CleanReads	Total MappingRatio	Uniquely MappingRatio	Multi MappingRatio	Discordantly MappingRatio
Mock-1	55390370	96.86%	88.59%	5.80%	2.47%
Mock-2	61411484	96.78%	88.75%	5.45%	2.58%
Mock-3	53927354	96.87%	88.64%	5.72%	2.51%
PR8-1	58119812	96.96%	89.35%	5.26%	2.35%
PR8-2	41423336	96.34%	88.35%	5.18%	2.81%
PR8-3	24550746	97.78%	90.51%	5.54%	1.73%
SBE-1	71933730	97.05%	88.96%	5.85%	2.24%
SBE-2	43859858	97.31%	89.45%	5.83%	2.03%
SBE-3	58825820	97.00%	89.01%	5.68%	2.31%

Mock-1, Mock-2, and Mock-3: three biological replicates of the normal cell samples; PR8-1, PR8-2 and PR8-3: three biological replicates of the PR8-infected samples; SBE-1, SBE-2, and SBE-3: three biological replicates of SBE treatment with PR8-infected samples.

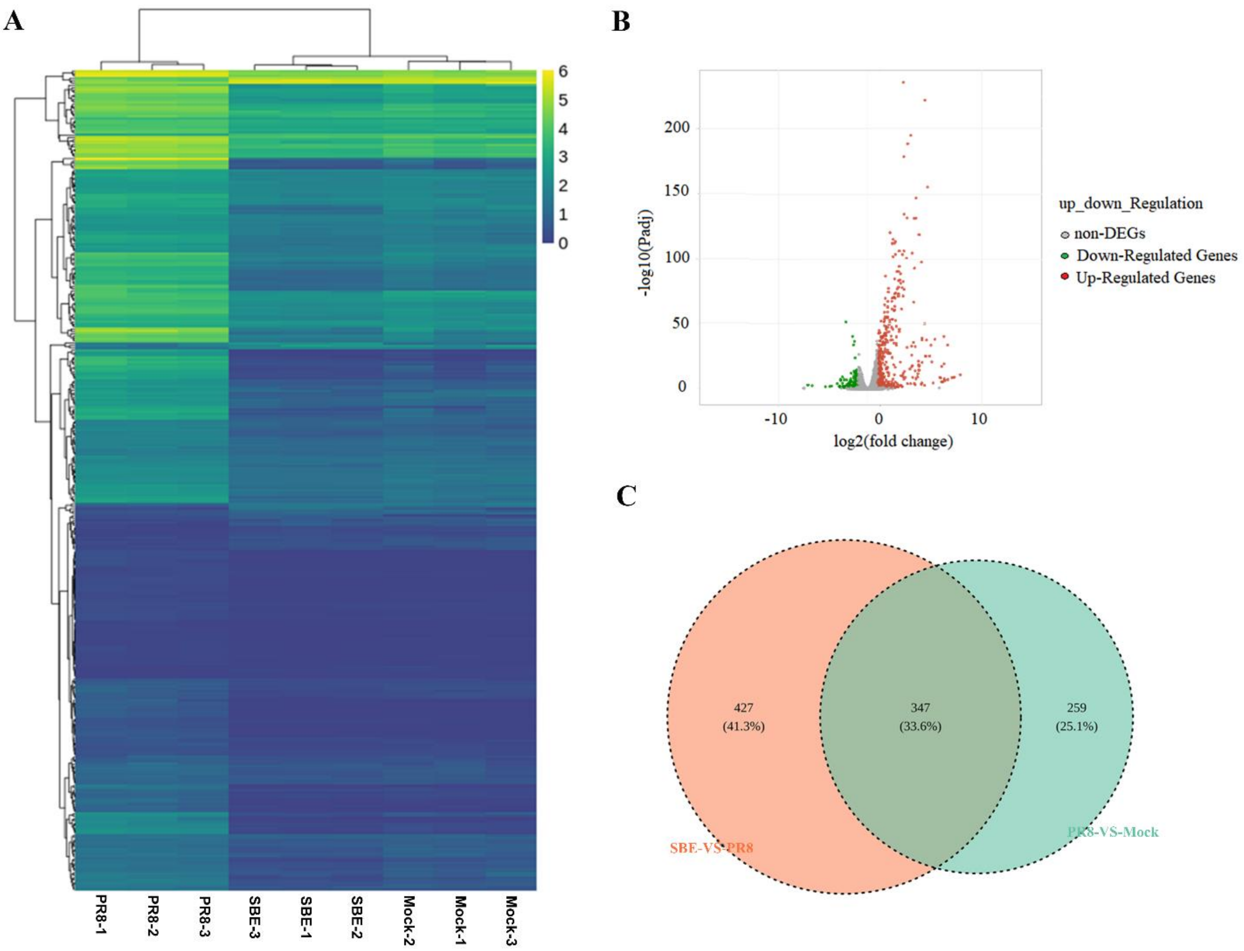

Supplementary Figure S1. Analysis of DEGs identified from the samples of Mock, PR8 and SBE groups. (**A**) Heatmap for cluster analysis of DEGs between Mock, PR8 and SBE groups. The union and inter DEGs were extracted from the results of three groups of differences, and FPKM values of each sample were used for cluster analysis to study the expression changes of DEGs in Mock, PR8 and SBE groups of samples. The color value of the heatmap is $\log_{10}$(FPKM+1), and the more yellow of the color represents the higher expression level. (**B**) Volcano plots of the distribution of DEGs between PR8 and Mock groups. The red, green and gray dots represent up-regulated, down-regulated and not significantly regulated genes, respectively. The X-axis indicates the Log_2 (fold change) of DEGs. The Y-axis represents the statistical significance, $-\mathrm{Log}_{10}$ (p-value). (**C**) Venn diagram shows the union and overlapping DEGs between Mock, PR8 and SBE groups.

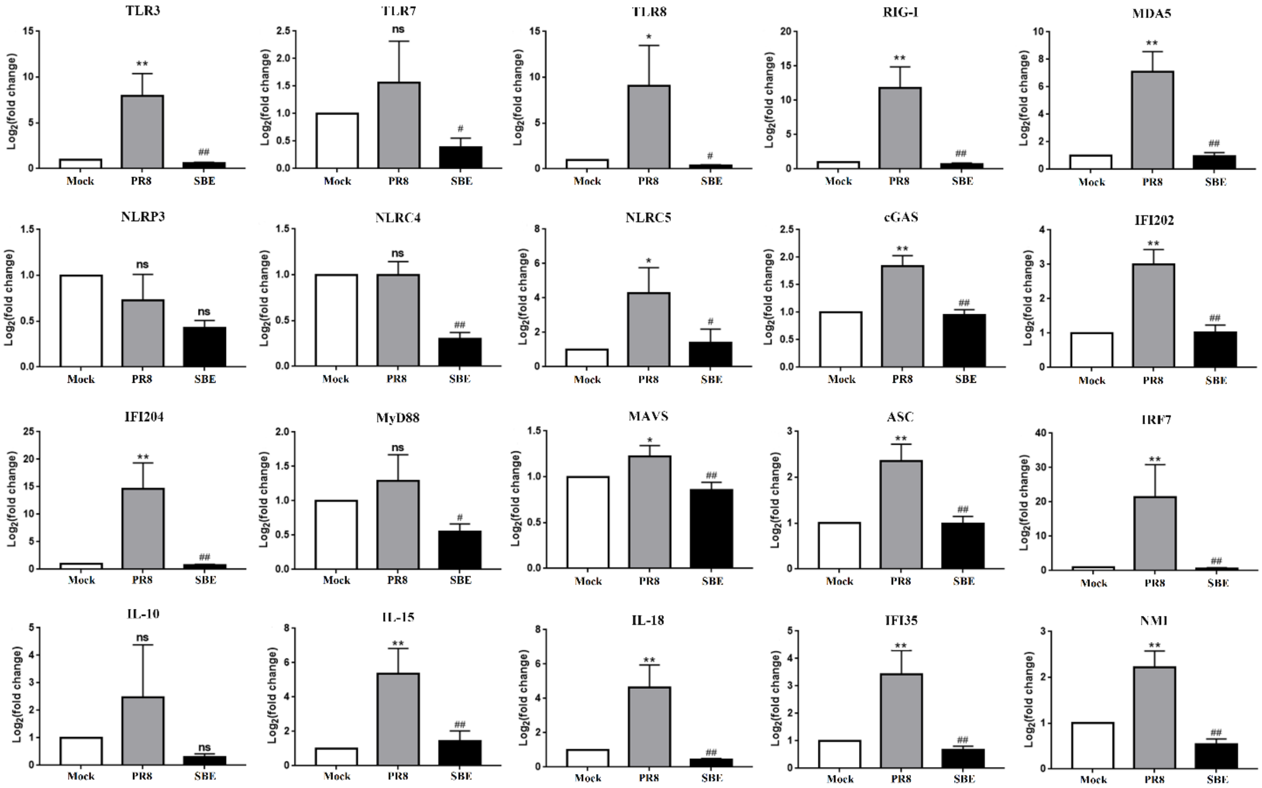

Supplementary Figure S2. The mRNA levels of representative DEGs involved in pattern recognition receptor pathways detected by RNA-Seq. The mRNA levels of pattern recognition receptors (TLR3, TLR7, TLR8, RIG-I, MDA5, NLRP3, NLRC4, NLRC5, cGAS, IFI202 and IFI204), adaptors (MyD88, MAVS, ASC), transcript factor IRF7 and downstream effect genes (IL-10, IL-15, IL-18, IFI35 and NMI) in Mock, PR8 and SBE groups were detected by RNA-seq. Data were obtained from three biological replicates. $^{*}P<0.05$, $^{**}P<0.01$ vs Mock group; $^{\#}P<0.05$, $^{\#\#}P<0.01$ vs PR8 group; ns indicates no statistical significance.
